# Multi-scale Gaussian product-of-experts for missing sequence multiple sclerosis lesion segmentation

**DOI:** 10.3389/fradi.2026.1868811

**Published:** 2026-09-29

**Authors:** Seung Hoon Lee, Lun Matthew Wong, Tiffany Y. So

**Affiliations:** Department of Imaging and Interventional Radiology, The Chinese University of Hong Kong, Shatin, Hong Kong SAR, China

**Keywords:** deep learning, magnetic resonance imaging, missing sequence, multiple sclerosis, segmentation

## Abstract

**Objective:**

To develop and evaluate a unified framework for multiple sclerosis (MS) white matter lesion segmentation that operates robustly under clinically prevalent missing sequence magnetic resonance imaging conditions.

**Methods:**

We propose a multi-scale Gaussian product-of-experts framework (MG-PoE) integrated into nnU-Net, where sequence-specific inputs are independently processed through a shared encoder and parameterized as Gaussian experts before combination through precision-weighted product-of-experts (PoE) fusion at each encoder scale. The unified fusion rule handles different sequence configurations without requiring subset-specific architectures. The framework was systematically evaluated under different sequence configurations on diverse datasets (MSSEG2016, MS3SEG, and MSLesSeg24).

**Results:**

On the five-sequence MSSEG2016, the proposed framework achieved the best overall performance compared to benchmark methods across all full-sequence and partially observed multisequence settings, with a mean Dice Similarity Coefficient (DSC) of 0.7029, a mean 95th percentile Hausdorff distance (HD95) of 22.4030, and a mean Composite Score (CS) of 0.7235 across the 26 sequence configurations. On MS3SEG, MG-PoE achieved the highest mean DSC and CS across the full sequence and missing-sequence settings, with significant improvements over competing methods, while HD95 performance was generally comparable. In zero-shot evaluation on MSLesSeg24, the method achieved the best overall DSC and HD95 in the full sequence setting (DSC, 0.5566; HD95, 27.3729) and the best mean DSC, HD95 and CS across the missing-sequence settings (DSC, 0.5096; HD95, 30.5054; CS, 0.5811).

**Conclusion:**

The MG-PoE framework provides a unified approach to missing-sequence MS lesion segmentation, achieving favorable performance on independent datasets and providing initial evidence of cross-dataset robustness.

## 1 Introduction

Multiple sclerosis (MS) is a chronic immune-mediated disease of the central nervous system characterized by demyelination, axonal injury, and progressive neurodegeneration, with formation of focal white matter lesions detectable on magnetic resonance imaging (MRI) (1, 2). Accurate and reproducible lesion segmentation is important for localizing MS lesions, characterizing their spatial distribution, and supporting objective radiological assessment (3–5). However, manual lesion delineation remains labor-intensive and subject to inter-rater variability, especially when lesions are small and numerous, motivating the development of automated MS lesion segmentation methods (6, 7).

Multisequence MRI, typically comprising T1-weighted (T1W), T2-weighted (T2W), fluid-attenuated inversion recovery (FLAIR), proton density (PD), and contrast-enhanced T1-weighted (T1CE) images, provides complementary tissue contrast that is valuable for automated lesion segmentation (8–11). Convolutional neural networks (CNNs), and more recently, transformer-based architectures have demonstrated strong performance when complete multisequence inputs are available at inference. In particular, nnU-Net has established itself as a robust and self-configuring baseline across a broad range of medical image segmentation tasks (12). Despite these advances, complete multisequence acquisitions are not always available in clinical practice because of protocol variation, patient motion, or image degradation affecting image quality (13). Missing sequences therefore pose a major challenge for robust MS lesion segmentation and model generalization, because models trained only on complete multisequence inputs tend to learn joint input statistics and inter-sequence dependencies specific to the complete-input setting. In joint-input architectures such as nnU-Net, sequence channels are mixed from the first convolution onward, so inter-sequence relationships are encoded at a very early stage of representation learning. Consequently, if one or more sequences are absent at inference time, performance can degrade because the test-time input distribution no longer matches the training condition (14, 15).

A practical strategy for addressing missing sequences is sequence-dropout training. ModDrop randomly removes one or more input sequences during training, exposing the network to different incomplete-input conditions (16). ModDrop++ extends this strategy by incorporating dynamic convolution and a structural similarity index measure to stabilize early feature representations across different-availability conditions (17). Although these dropout-based strategies broaden the input configurations seen during training, they do not explicitly weight the observed sequences according to their informativeness. As sequences are still combined within a joint-input network, the model must implicitly learn to accommodate different availability patterns within a single shared representation.

This reflects a related and more fundamental methodological challenge, in how information from the available sequences should be represented and integrated. Even when a model has been exposed to incomplete inputs during training, its ability to generalize depends on whether the fusion mechanism can make effective use of the heterogeneous evidence provided by the observed subset. Prior work has emphasized that accurate multisequence segmentation depends on effectively exploiting complementary information across sequences, and that different sequences may be sensitive to different region characteristics, implying inherently uneven contributions to segmentation (11).

Previously Havaei et al. proposed HeMIS, which preserves sequence-specific representations before latent aggregation, therefore moving away from direct input-level early fusion under missing-sequence conditions (18). In HeMIS, each imaging sequence is processed independently, and the resulting sequence-specific feature representations are aggregated using their mean and variance. Although HeMIS represents an early approach to incomplete sequence segmentation, its sequence-wise processing and statistical aggregation remains conceptually well suited to accommodate a range of missing sequences. Nevertheless, its aggregation rule is fixed and treats each extracted sequence representation equally, without estimating the reliability of the information provided by each sequence. A less informative or lower-quality sequence may be given the same weight as a more informative one, which can weaken the fused representation.

Task-conditional mixture-of-experts (TC-MoE) is another strategy that addresses missing sequences from a different perspective (19). Rather than maintaining independently modeled sequence representations until fusion, the available sequences are combined at the early stage, and a modality code is used to condition mixture-of-experts blocks. The model can therefore adapt its parameter combinations and computational pathway to the observed sequence configuration. In other words, TC-MoE handles missing sequences primarily through sequence-configuration-dependent routing and adaptive expert weighting within the encoder. This is effective for handling variable sequence availability, however, because the sequence information is mixed early, the contribution and uncertainty of each individual sequence is not retained and modeled throughout the feature hierarchy.

A probabilistic formulation of sequence fusion would be helpful to account for sequence-specific uncertainty but remains unexplored in the MS lesion segmentation literature. Existing fusion strategies typically either aggregate sequence representations through learned gating or routing mechanisms, where behavior depends on the observed input pattern, or summarize incomplete multisequence information without an explicit evidence-combination rule. This is a particular limitation in missing-sequence segmentation, where the observed sequence subset varies across cases. In such settings, a product-of-experts (PoE) is attractive because it provides a fusion rule that remains consistent across any observed subset, while explicitly weighting each sequence's contribution according to its estimated precision. A Gaussian product-of-experts formulation provides such a fusion rule. Each available sequence is represented as an individual Gaussian expert with its own estimated mean and variance, and the experts are combined according to their precisions. Consequently, sequences associated with lower predicted uncertainty contribute more strongly to the fused representation, whereas those associated with higher predicted uncertainty contribute less. Unlike the mean and variance aggregation in HeMIS, which produces a set-level summary of the available feature maps, Gaussian PoE directly uses sequence-specific precision to determine the contribution of each sequence during fusion.

In the classical PoE formulation, multiplying expert distributions combines their evidence into a joint distribution with accumulated precision (20). PoE-based multimodal inference has similarly shown that modality-specific evidence from arbitrary observed subsets can be combined into a consistent joint latent posterior (21). These properties make Gaussian PoE well suited to missing-sequence segmentation, where both the number and identity of the observed sequences can vary between cases. The same probabilistic fusion operation can be applied to any observed sequence subset.

In this study, we propose a multi-scale Gaussian product-of-experts framework (MG-PoE) for missing-sequence MS lesion segmentation. The proposed framework extends a self-configuring 3D nnU-Net by combining sequence-wise shared encoding with Gaussian precision-weighted PoE fusion at multiple encoder stages (12). Rather than concatenating all available sequences at the input, each sequence is processed separately using the same shared encoder, allowing sequence-specific feature representations to be retained until fusion. At each encoder stage, these representations are parameterized as Gaussian experts and combined according to their estimated precisions. The resulting fused representations are transferred to the decoder through the corresponding skip connections, while the deepest fused representation is propagated through the bottleneck. Thus, the decoder receives multisequence information that has already been integrated through the same probabilistic fusion rule at multiple resolutions. Sharing encoder weights across sequences avoids parameter increase and maintains a trainable parameter count comparable to that of the underlying nnU-Net. We systematically evaluated the framework under diverse sequence configurations on the MSSEG2016, MS3SEG, and MSLesSeg24 datasets.

## 2 Methods

### 2.1 Problem setting

Let $x=[x_{1},\ldots ,x_{M}]$ denote a multisequence MRI volume with *M* sequences and corresponding lesion label *y*. In missing-sequence segmentation, only a subset of sequences may be available at deployment time. During missing-sequence training, we represent the sequence-availability pattern by a binary vector $a=(a_{1},\ldots ,a_{M})\in \{0,1{\}}^{M}$ where $a_{m}=1$ indicates that sequence *m* is present. Our goal is to train a single segmentation model capable of handling sequence subsets containing at least two available sequences without requiring separate subset-specific models or modality imputation. Although single-sequence settings are also clinically relevant, they do not involve cross-sequence fusion, which focuses on combining complementary evidence from multiple available sequences. Therefore, we chose not to evaluate single-sequence segmentation, but rather to cover the incomplete acquisitions most commonly encountered in clinical practice.

A naive strategy for missing-sequence segmentation is to treat each sequence subset as a separate condition and learn a dedicated fusion pathway for that subset. For a multisequence MRI protocol with *M* sequences, the number of possible non-empty sequence subsets is $2^{M}-1$. In volumetric lesion segmentation, this combinatorial growth is problematic because each subset-specific branch must process dense multi-scale 3D features and support voxel-wise prediction. Consequently, subset-specific modeling would not only increase computational and memory cost during training and inference but also fragment the available training data across many sequence configurations. We therefore adopt a unified fusion rule parameterized by sequence availability, enabling a single model to handle the complete-input setting and the partially observed multisequence configurations containing at least two available sequences.

We realise this fusion rule in a unified segmentation framework built on nnU-Net. The overall architecture consists of a sequence-wise shared encoder, a scale-wise Gaussian product-of-experts fusion module, and a shared decoder, as illustrated in Figure 1. The encoder-decoder topology, including the network depth, feature-channel dimensions, convolutional kernels, and downsampling strides, was automatically determined by the corresponding nnU-Net plan. MG-PoE was then instantiated on the resulting backbone by applying Gaussian PoE fusion at every encoder scale defined by that plan. This design is also consistent with several prior incomplete multisequence segmentation studies, in the sense that multisequence information is integrated in the feature space rather than through direct early fusion (10, 18).

**Figure 1:**
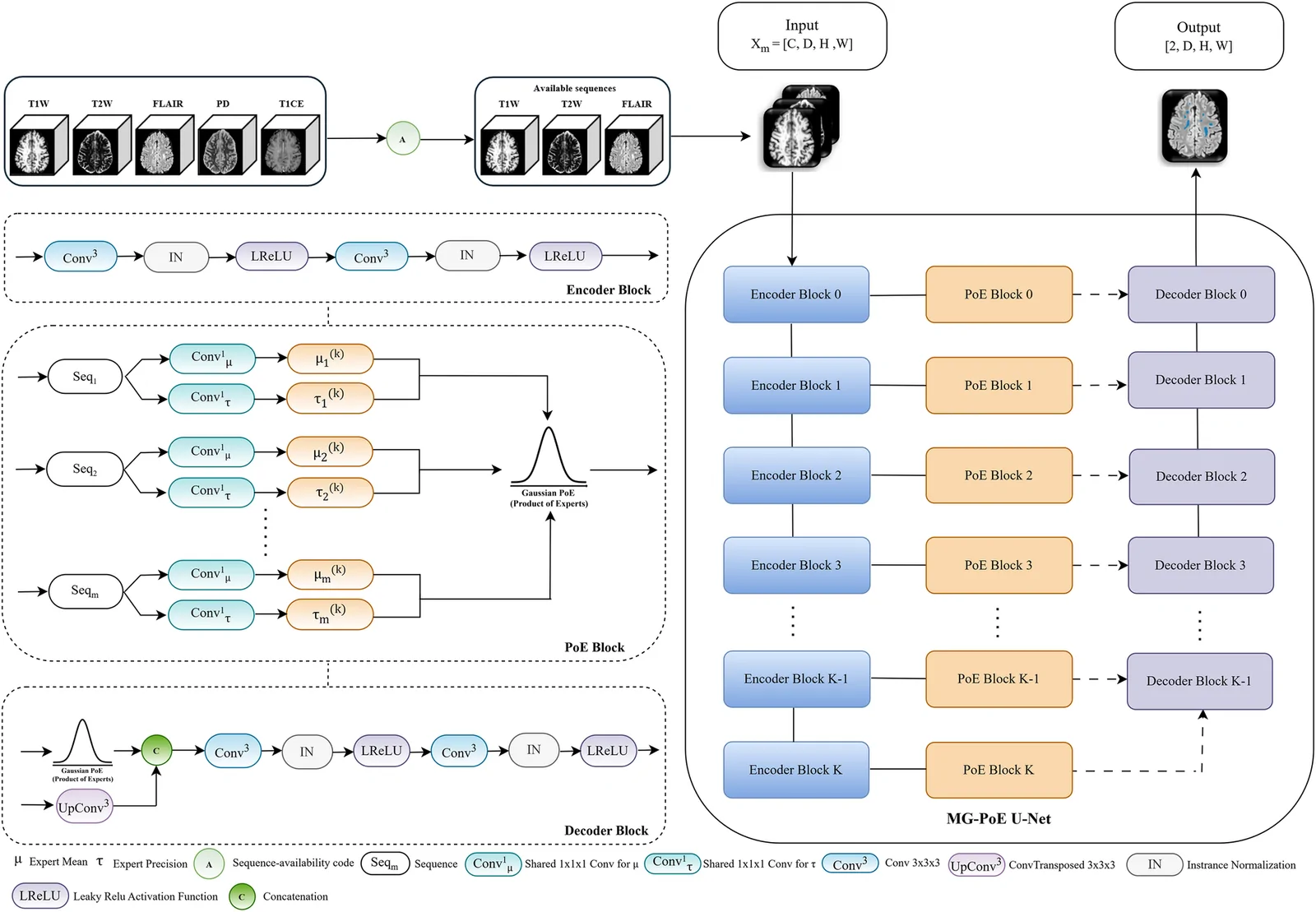
Overview of the proposed MG-PoE framework.

### 2.2 Scale-wise Gaussian product-of-experts fusion

At encoder scale k, we perform latent fusion using a Gaussian product-of-experts (PoE) formulation. As illustrated in Figure 1, rather than merging all sequences at a single stage, the proposed framework first extracts sequence-specific feature maps and then combines them through a shared latent fusion rule. This design is motivated by the missing-sequence setting, in which the observed sequence subset may vary across cases. Instead of relying on direct input-level combination, we model the contribution of each sequence as sequence-specific latent evidence about a common fused representation.

Let $z^{\left(k\right)}$ denote the target fused latent representation at encoder scale k, that is, the final scale-wise representation to be estimated from the observed sequence-specific features before being passed to the decoder. For notational simplicity, we write this latent variable as z when the scale k is fixed. For sequence m at scale k, we define a sequence-specific Gaussian expert

(1) $$\begin{array}{c} q_{m}^{\left(k\right)}(z|x_{m})=N(z;\mu_{m}^{\left(k\right)},\mathrm{diag}{\left(\tau_{m}^{\left(k\right)}\right)}^{-1}) \end{array}$$

where $\mu_{m}^{\left(k\right)}$ and $\tau_{m}^{\left(k\right)}$ denote the expert mean and element-wise precision, respectively. Equation (1) represents the sequence-specific latent distribution induced by sequence m, that is, how sequence m alone provides evidence about the target fused latent representation z at scale k. To make PoE fusion computationally tractable, we adopt the diagonal Gaussian parameterization in Equation (1) (21, 22).

Let $a_{m}\in \{0,1\}$ denote the sequence-availability indicator of sequence m, where $a_{m}$ = 1 if the sequence is observed and $a_{m}=0$ otherwise, and let $A=\{m:a_{m}=1\}\;$ denote the corresponding observed-sequence set. The sequence-specific experts are then combined through a PoE rule together with a fixed prior expert,

(2) $$\begin{array}{c} q_{\mathrm{PoE}}^{\left(k\right)}(z|x_{A})\propto p_{0}(z)\overset{M}{\underset{m=1}{\prod }}\;{\left[q_{m}^{\left(k\right)}(z|x_{m})\right]}^{a_{m}} \end{array}$$

where $p_{0}(z)$ is a zero-mean spherical Gaussian prior expert. Because the exponent $a_{m}$ is zero for missing sequences, unavailable experts are excluded from the product automatically, allowing the same fusion rule to handle arbitrary missing-sequence subsets without introducing separate fusion pathways.

Under this assumption, the product in Equation (2) remains Gaussian, so the fused posterior distribution can be written in closed form. In the present segmentation setting, we use the corresponding posterior mean as the fused latent representation.

(3) $$\begin{array}{c} \mu_{\mathrm{PoE}}^{\left(k\right)}=\frac{\sum_{m=1}^{M}\;a_{m}\;\tau_{m}^{\left(k\right)}\;\mu_{m}^{\left(k\right)}}{\tau_{0}+\sum_{m=1}^{M}\;a_{m}\;\tau_{m}^{\left(k\right)}} \end{array}$$

Equation (3) shows that the fused latent representation is given by a precision-weighted combination of the sequence-specific latent evidence. Since precision is the reciprocal of variance, experts with higher precision, that is, lower uncertainty, contribute more strongly to the fused result.

In our implementation, as shown by the two shared 1 × 1 × 1 convolution branches, the encoder feature map $h_{m}^{\left(k\right)}$ is used to predict the parameters of the Gaussian expert

(4) $$\begin{array}{c} \mu_{m}^{\left(k\right)}=f_{\mu }^{\left(k\right)}(h_{m}^{\left(k\right)}),\;\;\tau_{m}^{\left(k\right)}=f_{\tau }^{\left(k\right)}(h_{m}^{\left(k\right)}) \end{array}$$

where $f_{\mu }^{\left(k\right)}(\cdot )$ and $f_{\tau }^{\left(k\right)}(\cdot )$ are learnable mappings. These lightweight 1 × 1 × 1 heads parameterize the Gaussian expert in Equation (1), whereas the actual cross-sequence fusion is performed by the PoE rule in Equation (2) and the closed-form posterior mean in Equation (3). In the present segmentation setting, we do not use the full posterior for generative reconstruction; instead, we use the posterior mean $\mu_{\mathrm{PoE}}^{\left(k\right)}$ as the fused latent feature at scale k and pass it to the decoder. The same Gaussian PoE rule is applied independently at every encoder scale, yielding a unified multi-scale fusion mechanism for incomplete multisequence inputs.

### 2.3 Decoder

The fused multi-scale features $\{z^{\left(0\right)},\ldots ,z^{\left(K\right)}\}\;$ are provided to a shared decoder through the bottleneck and corresponding skip connections to generate lesion segmentation outputs. The decoder follows a U-shaped design with transposed convolutions for upsampling and skip connections from the fused encoder features. At each stage, the upsampled feature map is concatenated with the corresponding fused feature and refined by convolutional blocks. As illustrated in Figure 1, this design allows the decoder to leverage PoE-fused representations at multiple semantic levels while preserving the standard hierarchical reconstruction pathway of U-Net (23).

### 2.4 Training under simulated missing sequence settings

During training, missing-sequence settings are simulated by randomly masking one or more sequences while ensuring that at least one sequence remains available. In addition to the segmentation loss $L_{\mathrm{seg}}$, we use a weak auxiliary regularizer that discourages fusion-weight collapse onto a single available sequence:

(5) $$\begin{array}{c} L_{\mathrm{total}}=L_{\mathrm{seg}}+\lambda_{\mathrm{aux}}L_{\mathrm{aux}} \end{array}$$

Here, $L_{\mathrm{aux}}$ is computed as the average squared deviation between the normalized mean-precision contribution of each available sequence and the corresponding available-only uniform target, averaged over fusion scales and valid samples. This design is motivated by the fact that, in precision-weighted multisequence fusion, an excessively large weight can cause one sequence to dominate the fused representation. Our auxiliary term therefore does not strictly enforce equal weighting across sequences, but instead softly stabilizes training by discouraging degenerate dominance while preserving adaptive fusion.

## 3 Experiments

### 3.1 Datasets

Experiments were conducted on three public MS lesion segmentation datasets, MSSEG2016, MS3SEG, and MSLesSeg24 **(**Table 1**)** (24–26). For all datasets, the original dataset-provided ground-truth lesion masks were used according to their respective annotation protocols.

**Table 1:** Overview of datasets used in this study.

Dataset	Cases/series	Sequences	Scanner	Tesla
MSSEG2016	53 patients	T1W, T2W, FLAIR, PD, T1CE	Siemens/Philips/GE	1.5 T, 3.0 T
MS3SEG	100 patients	T1W, T2W, FLAIR	Toshiba	1.5 T
MSLesSeg24	75 patients/115 series	T1W, T2W, FLAIR	multi-scanner	1.5 T, 3.0 T

MSSEG2016 served as the primary benchmark for the main method comparison and component-wise ablation. This dataset contains 53 patients, comprising 15 training cases and 38 test cases, with five MRI sequences available for each case: T1W, T2W, FLAIR, PD, and T1CE. The data were acquired across multiple centers using different scanner vendors and field strengths under a harmonized acquisition protocol. As part of the dataset-provided preprocessing, the MRI sequences underwent denoising and rigid registration to the FLAIR reference geometry. An intracranial mask generated from the native T1W image was transformed into the common FLAIR space and applied to the registered sequences for skull stripping, followed by bias-field correction. Training was performed on the official MSSEG2016 training set and evaluated on the test set.

MS3SEG comprised 100 patients, each contributing a single MRI session acquired using a 1.5T Toshiba Vantage scanner. The dataset provided axial T1W, T2W, and FLAIR images. During dataset preprocessing, the T1W and T2W images were registered to the axial FLAIR. Brain extraction was performed on the FLAIR image, and the resulting brain mask was applied to the registered T1W and T2W images. MS3SEG was used as a secondary benchmark, with training and evaluation performed using the subject-level three-fold cross-validation.

MSLesSeg24 contains 75 patients and 115 MRI series, including 53 patients in the original training partition and 22 patients in the original test partition, and provides three MRI sequences, T1W, T2W and FLAIR, in a longitudinal and clinically heterogeneous setting. The images originated from different 1.5T and 3.0T scanners and were not acquired using a single uniform protocol. During dataset preprocessing, each MRI sequence was registered to the 1-mm isotropic MNI152 template, followed by brain extraction. MSLesSeg24 was used for zero-shot evaluation of models trained exclusively on MSSEG2016. The available T1W, T2W, and FLAIR images were mapped to their corresponding model input channels, whereas PD and T1CE were treated as unavailable sequences. Because neither the original MSLesSeg24 training partition nor the test partition was used for model development, fine-tuning, or model selection, the two partitions were combined to increase the size of the independent zero-shot evaluation cohort without introducing overlap with the MSSEG2016 development data. The resulting analysis included all 115 MRI series, with baseline and longitudinal scans treated as individual evaluation samples. Accordingly, the reported results represent descriptive scan-level performance on the full available MSLesSeg24 cohort rather than performance restricted to its official held-out test partition.

### 3.2 Implementation details

All models were implemented in PyTorch and executed within the nnU-Net framework (12, 27). Experiments were performed on NVIDIA A100 GPUs with 80 GB memory. Following the standard nnU-Net procedure, each case was first cropped to its non-zero region to remove redundant background. Intensity normalization was performed independently for each image using z-score normalization, consistent with standard nnU-Net practice for MRI data.

Across all methods, preprocessing, data augmentation, normalization, missing-sequence input construction, and evaluation procedures were harmonized to reduce preprocessing-related confounding.

Patch-based sliding-window prediction was performed using the patch size adopted during training for each method, with 50% overlap between adjacent windows and Gaussian importance weighting to aggregate overlapping predictions.

For MG-PoE, the network depth, feature-channel schedule, convolutional kernel sizes, downsampling strides, and number of convolutional blocks were inherited from the corresponding dataset-specific nnU-Net plans rather than manually selected. The sequence-wise shared encoding architecture and multi-scale Gaussian PoE fusion formulation remained unchanged across the independently trained datasets.

For both independently trained datasets, MG-PoE was optimized using stochastic gradient descent with Nesterov momentum, an initial learning rate of $1\times 10^{-2}$, a weight decay of $3\times 10^{-5}$, and a polynomial learning-rate decay schedule. Training was conducted for 100 epochs in two phases. During the first phase, the model was trained for 40 epochs using complete multisequence inputs to stabilize sequence-wise representation learning before introducing missing-sequence simulation. During the second phase, training continued for 60 epochs under randomly simulated missing-sequence conditions. For each training sample, a random non-empty subset of the available sequences was selected, including occasional single-sequence inputs, to expose the model to a broad range of sequence-availability conditions. During this training phase, sequence-availability indicators were provided to the PoE fusion modules to exclude unavailable sequence experts from the fusion process. The auxiliary dominance regularization weight was set to $\lambda_{\mathrm{aux}}=2\times 10^{-4}$.

For MSSEG2016, MG-PoE used the dataset-specific 3D full-resolution configuration with a patch size of 96 × 160 × 160 and a batch size of 2. For MS3SEG, the corresponding 3D full-resolution nnU-Net plan specified a patch size of 24 × 256 × 256 and a batch size of 2.

### 3.3 Benchmarking

MG-PoE was compared with HeMIS, ModDrop++, and TC-MoE (17–19). To ensure fair comparison, preprocessing, data augmentation, missing-sequence input construction, and evaluation procedures were harmonized across methods wherever applicable. The original architecture and method-specific training strategies of the benchmark methods were otherwise preserved as closely as possible. HeMIS was extended from 2D to 3D by replacing its convolutional and pooling operations with their volumetric counterparts, while ModDrop++ and TC-MoE retained their original model structures and optimization strategies. Detailed implementation and training configurations are provided in the Supplementary Table S1.

### 3.4 Evaluation metrics

Segmentation performance was assessed using the Dice Similarity Coefficient (DSC) to quantify volumetric overlap between the predicted and reference segmentations, and the 95th percentile Hausdorff Distance (HD95) to assess boundary-level agreement while reducing sensitivity to outliers relative to the standard Hausdorff Distance (28). As MRI-derived lesion metrics, including lesion count, are also clinically important in multiple sclerosis, evaluation should capture not only voxel-wise overlap but also lesion detection (29). Furthermore, MS lesion segmentation is intrinsically challenging because lesion appearance is heterogeneous, lesion voxels constitute only a small fraction of the total brain volume, and DSC can be dominated by larger lesions.

To account for this, and consistent with prior literature, we further report the composite score (CS) (17, 30, 31), defined as:

(6) $$\begin{array}{c} \;CS\;=\;\frac{DSC}{8}\;+\;\frac{PPV}{8}\;+\;\frac{1-LFPR}{4}+\frac{LTPR}{4}+\frac{Cor}{4} \end{array}$$

In addition to DSC, the CS incorporates positive predictive value (PPV), which measures voxel-wise precision; lesion-wise true positive rate (LTPR), calculated as the proportion of detected reference lesions which overlapped the predicted segmentation by at least one voxel; lesion-wise false positive rate (LFPR), calculated as the proportion of false-positive predicted lesions; and the correlation term (Cor), defined as the case-wise voxel-wise Pearson correlation between the flattened binary predicted and the reference segmentation masks. The metric values were averaged across cases for each sequence configuration. Case-level paired comparisons were performed separately within each sequence configuration, and configurations were subsequently pooled to obtain the reported averages.

### 3.5 Ablation study design

All ablation configurations were instantiated using the same MSSEG2016 nnU-Net plan. Accordingly, the stage-wise feature channel schedule, convolutional kernels, downsampling strides, patch size, preprocessing, and training protocol were held constant across variants, while only the encoder structure, the fusion mechanism, and auxiliary regularization were varied. The ablation study was not intended as a fully factorial decomposition because some sequence-processing and fusion choices are structurally incompatible and therefore cannot be evaluated independently.

In the standard joint-input nnU-Net baseline, all available sequences are concatenated at the input and processed jointly by a single encoder. Consequently, cross-sequence interaction begins at the first convolution, and the individual sequences are not represented separately throughout the encoder. By contrast, sequence-wise processing encodes each available sequence separately and retains the resulting sequence-specific representations until fusion. Within the sequence-wise framework, we varied both the encoder parameterization and the fusion rule. Sequence-wise encoding was implemented using either independently parameterized sequence-specific encoders or a shared encoder whose weights were reused across sequences, while the resulting sequence-specific features were combined using either mean fusion or PoE fusion.

The ablation series comprised several separate configurations. The first configuration was the standard joint-input nnU-Net baseline. The two subsequent configurations replaced joint-input processing with independently parameterized sequence-specific encoders followed by fusion, thereby assessing the effect of preserving separate sequence representations before fusion. The next configuration replaced the independently parameterized encoders with a shared encoder, allowing the effect of encoder weight sharing to be evaluated. The subsequent configuration retained the shared encoder and compared mean fusion with multi-scale PoE fusion, without applying auxiliary dominance regularization. Finally, the auxiliary dominance regularizer was added to the shared-encoder, multi-scale PoE configuration to form the complete MG-PoE model. This final comparison assessed the contribution of dominance control. As the regularizer directly constrains the PoE weights to prevent the PoE fusion weights from becoming excessively concentrated on a single sequence expert, it was evaluated as part of the MG-PoE framework.

## 4 Results

### 4.1 Quantitative comparison on complete and partially observed multisequence settings

The quantitative results on MSSEG2016 are summarized in Table 2 and Supplementary Table S2. Compared with all baseline models, MG-PoE achieved favorable mean DSC and CS results while showing generally comparable HD95 Performance across most of the complete and missing-sequence configurations. Results on MS3SEG are presented in Table 3 and Supplementary Table S3. In both the full-sequence and missing-sequence settings, MG-PoE achieved the highest DSC and CS, while its HD95 remained broadly comparable to those of the baseline methods.

**Table 2:** Quantitative comparison on MSSEG2016.

Group	No. configurations	DSC				HD95				CS			
		HeMIS	ModDrop++	TC-MoE	MG-PoE	HeMIS	ModDrop++	TC-MoE	MG-PoE	HeMIS	ModDrop++	TC-MoE	MG-PoE
Full Sequence	1	0.7071	0.6781	0.7270	**0**.**7541**	22.0379	22.3431	20.3500	**18**.**8818**	0.6968	0.6819	0.7497	**0**.**7810**
Missing 1	5	0.6738	0.6522	0.7166	**0**.**7388**	23.4241	22.7254	22.3697	**20**.**4556**	0.6633	0.6562	0.7392	**0**.**7583**
Missing 2	10	0.5796	0.6190	0.6902	**0**.**7144**	26.2219	22.4406	23.2025	**21**.**4902**	0.5722	0.6252	0.7116	**0**.**7306**
Missing 3	10	0.3692	0.5761	0.6091	**0**.**6684**	30.9586	**22**.**0598**	27.2892	24.6417	0.4004	0.5859	0.6674	**0**.**6933**

Bold values indicate the best performance for the sequence-availability setting.

**Table 3:** Quantitative comparison on MS3SEG.

**Group**	**No. configurations**	**DSC**				**HD95**				**CS**			
		**HeMIS**	**ModDrop++**	**TC-MoE**	**MG-PoE**	**HeMIS**	**ModDrop++**	**TC-MoE**	**MG-PoE**	**HeMIS**	**ModDrop++**	**TC-MoE**	**MG-PoE**
Full Sequence	1	0.6962	0.7360	0.7449	**0**.**7850**	**21**.**8544**	24.1704	22.0122	23.6660	0.7036	0.7316	0.7518	**0**.**8141**
Missing 1	3	0.5966	0.6643	0.6667	**0**.**7175**	**23**.**6184**	24.0001	24.5032	24.2609	0.6080	0.6702	0.6759	**0**.**7508**

Bold values indicate the best performance for the sequence-availability setting.

Results on MSLesSeg24 are presented in Table 4 and Supplementary Table S4**.** In the full-sequence setting, MG-PoE achieved the highest DSC and lowest HD95, while its CS was slightly lower than that of TC-MoE. Under the missing-sequence settings, MG-PoE achieved the highest overall DSC and CS and the lowest HD95 among the evaluated methods. Absolute performance was generally lower than that observed on MSSEG2016 and MS3SEG, which may reflect differences in cohort characteristics, acquisition protocols and available MRI sequences.

**Table 4:** Quantitative comparison on MSLesSeg24.

Group	No. configurations	DSC				HD95				CS			
		HeMIS	ModDrop++	TC-MoE	MG-PoE	HeMIS	ModDrop++	TC-MoE	MG-PoE	HeMIS	ModDrop++	TC-MoE	MG-PoE
Full Sequence	1	0.3971	0.4512	0.5513	**0**.**5566**	41.6586	39.5756	32.8627	**27**.**3729**	0.4227	0.4884	**0**.**6355**	0.6180
Missing 1	3	0.2999	0.3805	0.4917	**0**.**5096**	43.0418	37.4491	34.5943	**30**.**5054**	0.3422	0.4338	0.5570	**0**.**5811**

Bold values indicate the best performance for the sequence-availability setting.

### 4.2 Ablation results

In the ablation experiments, replacing the single joint-input encoder of nnU-Net with sequence-specific encoders followed by mean fusion did not improve performance. The standard joint-input nnU-Net achieved higher DSC and CS and lower HD95 (Table 5), indicating that simply processing the sequences separately does not necessarily improve segmentation performance when each sequence is handled by independently parameterized encoders. However, under the same sequence-wise fusion setting, replacing the sequence-specific encoders with a shared encoder improved DSC, HD95, and CS, demonstrating the benefit of encoder weight sharing.

**Table 5:** Staged component-wise ablation on MSSEG2016.

**Encoder**			**Fusion**			**Loss Function**	**Performance**		
**Joint input encoder**	**Seq-wise specific encoders**	**Seq-wise shared encoder**	**Early**	**Mean**	**PoE**	**L_reg_**	**DSC**	**HD95**	**CS**
✓			✓				0.6846	**21**.**8090**	0.7057
	✓			✓			0.6280	26.5139	0.6833
	✓				✓		0.6407	25.4078	0.6904
		✓		✓			0.6655	24.5671	0.7016
		✓			✓		0.6849	21.9536	0.7119
		✓			✓	✓	**0**.**7029**	22.4030	**0**.**7235**

Bold values indicate the best performance for the sequence-availability setting.

With the shared sequence-wise encoder retained, replacing mean fusion with PoE fusion improved DSC and CS, indicating that precision-weighted fusion provided a more effective means of integrating sequence-specific evidence. Finally, adding the auxiliary dominance regularizer to the PoE-based configuration produced additional improvements in DSC and CS. Although the complete MG-PoE model achieved an HD95 comparable to that of the joint-input nnU-Net, it yielded significantly higher DSC and CS across the matched sequence-availability settings.

### 4.3 Qualitative comparison

Figure 2 presents representative missing-sequence scenarios. In this example, when only FLAIR was unavailable, MG-PoE and ModDrop++ generally showed visually similar results, with both methods preserving the principal lesion regions and showing relatively limited false-negative areas compared to HeMIS and TC-MoE. However, when additional sequences, including T1W and/or T2W, were removed together with FLAIR, HeMIS, ModDrop++ and TC-MoE showed more false-negative regions and greater configuration-dependent changes in lesion segmentation. In contrast, MG-PoE continued to preserve the major lesion outline and maintained comparatively consistent spatial overlap with the ground truth across the evaluated configurations. Findings show that MG-PoE may provide relatively stable lesion predictions despite the progressive loss of clinically informative MRI sequences.

**Figure 2:**
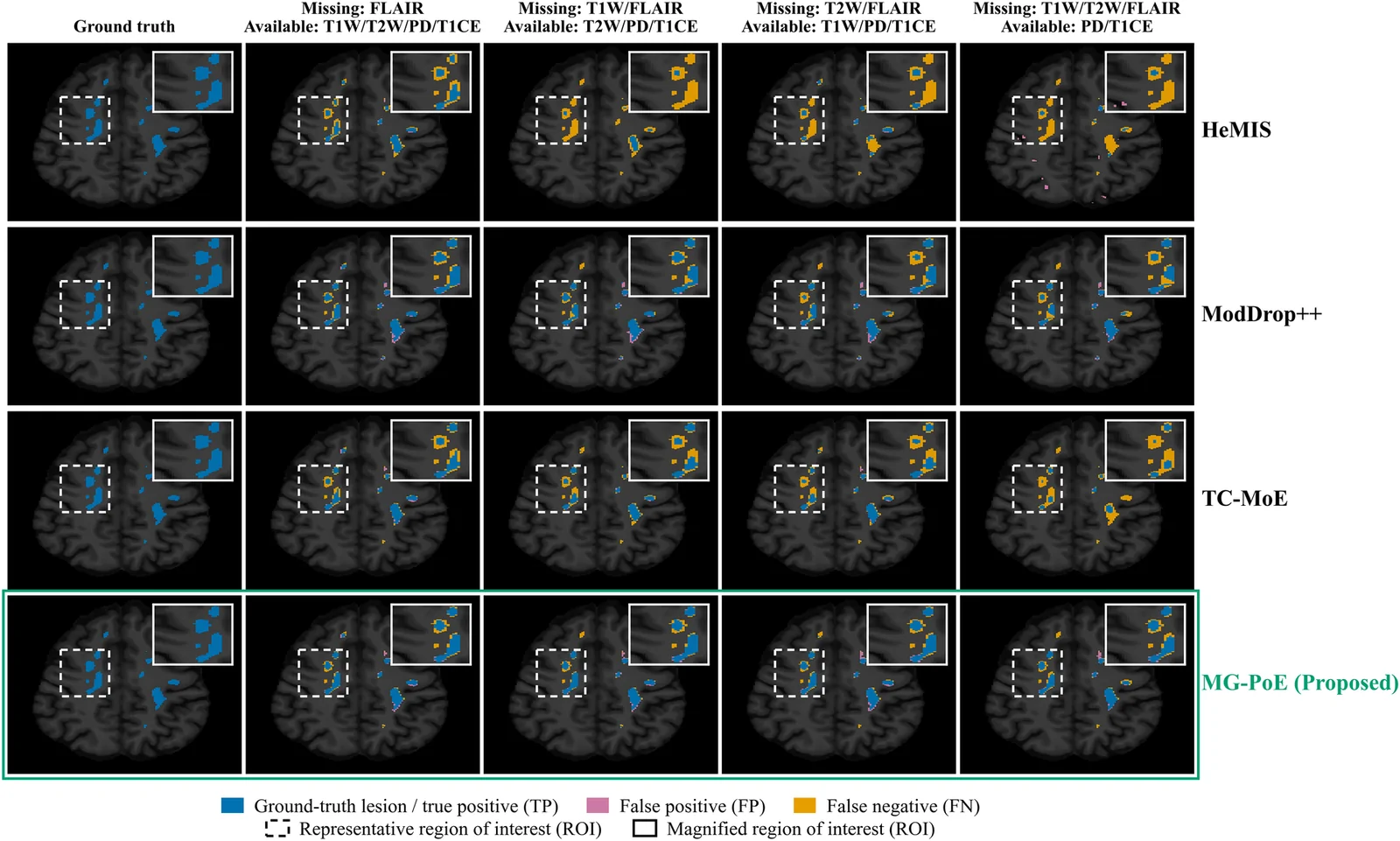
Representative qualitative comparisons among HeMIS, ModDrop++, TC-MoE, and MG-PoE under selected missing-sequence configurations. For clarity, only representative subsets are shown, with emphasis on missing T1W, T2W, and FLAIR settings. When only FLAIR is unavailable, the methods show relatively visually similar results, whereas clearer differences emerge as additional sequences are missing. MG-PoE preserves a more consistent segmentation performance despite the progressive loss of informative sequences.

## 5 Discussion

This study introduced MG-PoE, a multi-scale Gaussian product-of-experts framework for missing-sequence MS lesion segmentation, and systematically evaluated it across complete and partially observed multisequence configurations.

Accurate MS lesion segmentation is integral to patient assessment and disease monitoring, as total lesion burden and lesion count constitute established markers of disease severity. In longitudinal follow-up, reliable quantification of lesion-volume change is essential for evaluating treatment response and detecting subclinical progression. Automated segmentation methods that deliver consistent, reproducible measurements therefore hold substantial clinical value for both routine MS management and clinical trials. In practice, however, complete multisequence MRI is not always available, which limits the applicability of methods that require a fixed set of inputs. In this study, the results demonstrate the potential of MG-PoE to tolerate missing inputs while preserving and probabilistically combining sequence-specific information, therefore having potential to support reliable segmentation under incomplete acquisition protocols commonly encountered in clinical practice.

On the independent MSSEG2016 and MS3SEG datasets, MG-PoE showed favorable overall performance in both complete and missing-sequence settings. On MSSEG2016, its advantage was observed across a broad range of partially observed sequence subsets rather than being limited to selected configurations. On MS3SEG, MG-PoE achieved the highest overall DSC and CS in both the complete-sequence and missing-sequence settings, demonstrating favorable volumetric overlap and composite segmentation performance under a different three-sequence setting.

In the zero-shot evaluation on MSLesSeg24, the absolute performance of all methods was generally lower than that of MSSEG2016 and MS3SEG. The overall reduction in performance may reflect the underlying domain shift. However, the model nonetheless maintained competitive segmentation accuracy without any fine-tuning or adaptation to the target domain. Compared with the benchmarking models, MG-PoE achieved the highest DSC and lowest HD95 in the complete-sequence setting, and the highest mean DSC and lowest mean HD95 across the missing-sequence settings. MG-PoE also achieved the highest mean CS across the missing-sequence settings. These results can be interpreted as encouraging evidence of zero-shot out-of-domain generalization under restricted sequence availability. At the same time, because the evaluation was conducted on a limited range of sequence configurations, they should be regarded as an initial demonstration rather than comprehensive evidence of generalizability.

The incremental ablation results indicated that the main performance improvement arose from combining sequence-wise processing, shared encoder parameterization, and PoE fusion. Thus, the benefit of MG-PoE can be attributed to preserving sequence-specific evidence, leveraging a shared encoder, and integrating the available evidence through precision-weighted probabilistic fusion.

From a clinical perspective, the favorable CS performance of MG-PoE reflects multiple aspects of segmentation performance beyond voxel-level overlap, as it reflects both lesion detection and false-positive lesion control.

This study has several limitations. Firstly, only configurations containing at least two available sequences were evaluated because the proposed formulation focuses on statistical estimation and fusion among multiple sequence-specific representations. Single-sequence settings may be an important focus of investigation in future work. Secondly, the lower absolute performance on MSLesSeg24 suggests the need to further improve robustness to heterogeneous external domains. Finally, the available MRI sequences, acquisition protocols, and preprocessing procedures differed across datasets, limiting direct cross-dataset comparisons, although such differences reflect the heterogeneity encountered in real-world clinical imaging. Future studies should therefore validate the framework in larger multicenter cohorts covering a wider range of scanners and acquisition protocols.

## 6 Conclusion

This study introduced MG-PoE, a multi-scale Gaussian product-of-experts framework for robust missing-sequence MS lesion segmentation. MG-PoE combines sequence-wise shared encoding with scale-wise PoE fusion, preserving sequence-specific evidence before fusion and integrating the observed multisequence subsets through a unified precision-weighted combination fusion rule.

MG-PoE offers a promising and effective approach for missing-sequence MS lesion segmentation when more than one complementary sequence is present.

## Data Availability

The MSSEG2016 dataset is available through the MICCAI 2016 MSSEG Challenge data portal: https://portal.fli-iam.irisa.fr/msseg-challenge/. The MS3SEG dataset is available through Figshare: https://doi.org/10.6084/m9.figshare.30393475 and was released under CC BY 4.0. The MSLesSeg24 dataset is available through Figshare: https://doi.org/10.6084/m9.figshare.27919209 and was released under CC BY 4.0.
