# Supplementary material for: Multi-scale Gaussian product-of-experts for missing sequence multiple sclerosis lesion segmentation
Source: Front Radiol. 2026 Sep 29;6:1868811. doi: 10.3389/fradi.2026.1868811 (PMC13658566; doi:10.3389/fradi.2026.1868811)
Supplement: Supplementary file 1 [file Supplementaryfile1.docx]

**Supplementary Table s1. Baseline implementation details of benchmarking methods**

| **Baseline** | **Citation** | **Architecture / dimensionality** | **Patch size and batch size** | **Training schedule** | **Optimizer / learning-rate schedule /** |
| --- | --- | --- | --- | --- | --- |
| **HeMIS** | - Havaei M, Guizard N, Chapados N, Bengio Y. Hemis: Hetero-modal image segmentation. International Conference on Medical Image Computing and Computer-Assisted Intervention. (2016). p. 469–477 | - Volumetric 3D adaptation; original 2D convolution and pooling operations replaced by 3D counterparts. | - MSSEG2016: 96 × 160 × 160, batch 2 - MS3SEG: 24 × 256 × 256, batch 2 | - 100 epochs - 40 complete-input epochs + 60 random-missing epochs | - Nesterov momentum - Learning rate of 1×10^−2^   with polynomial decay. |
| **TC-MoE** | - Novosad P, Carano RA, Krishnan AP. A task-conditional mixture-of-experts model for missing modality segmentation. International Conference on Medical Image Computing and Computer-Assisted Intervention. (2024). p. 34–43 | - Original architecture retained; 3D full-resolution input. | - MSSEG2016: 96 × 160 × 160, batch 2 - MS3SEG: 24 × 256 × 256, batch 2 | - Original single-stage training protocol retained; - 100 epochs | - Adam optimizer - Learning rate 1e^−4^ with cosine decay |
| **ModDrop++** | - Liu H, Fan Y, Li H, Wang J, Hu D, Cui C, et al. Moddrop++: A dynamic filter network with intra-subject co-training for multiple sclerosis lesion segmentation with missing modalities. | - Original 2.5D architecture and input construction retained. | - Original 2.5D patch, batch 16 | - Original single-stage training protocol retained; - 300 epochs | - Adam optimizer with a momentum of 0.5 - Learning rate as 0.25 (linearly decayed after 100 epochs) |
