# Supplementary material for: Multi-scale Gaussian product-of-experts for missing sequence multiple sclerosis lesion segmentation
Source: Front Radiol. 2026 Sep 29;6:1868811. doi: 10.3389/fradi.2026.1868811 (PMC13658566; doi:10.3389/fradi.2026.1868811)
Supplement: Supplementary file 2 [file Table2.docx]

**Supplementary Table s2. Full quantitative results on MSSEG-2016**

| **Group** | **Available sequences** | | | | | **DSC** | | | | **HD95** | | | | **CS** | | | |
| --- | --- | --- | --- | --- | --- | --- | --- | --- | --- | --- | --- | --- | --- | --- | --- | --- | --- |
|  | **T1** | **T2** | **FLAIR** | **PD** | **T1CE** | **HeMIS** | **ModDrop++** | **TC-MoE** | **MG-PoE** | **HeMIS** | **ModDrop++** | **TC-MoE** | **MG-PoE** | **HeMIS** | **ModDrop++** | **TC-MoE** | **MG-PoE** |
| **Full Sequence** | ● | ● | ● | ● | ● | 0.7071 | 0.6781 | 0.7270 | **0.7541** | 22.0379 | 22.3431 | 20.3500 | **18.8818** | 0.6968 | 0.6819 | 0.7497 | **0.7810** |
| **Missing 1** | ● | ● | ● | ● |  | 0.7006 | 0.6660 | 0.7365 | **0.7539** | 21.7751 | 22.9532 | 21.9212 | **19.9721** | 0.6955 | 0.6727 | 0.7524 | **0.7757** |
|  | ● | ● | ● |  | ● | 0.7056 | 0.6659 | 0.7313 | **0.7536** | 23.7617 | 23.4589 | 21.9798 | **19.1486** | 0.6986 | 0.6768 | 0.7527 | **0.7763** |
|  | ● | ● |  | ● | ● | 0.5771 | 0.5885 | 0.6503 | **0.6745** | 25.0461 | **22.1560** | 24.2036 | 24.1760 | 0.5713 | 0.5783 | 0.6742 | **0.6848** |
|  | ● |  | ● | ● | ● | 0.6834 | 0.6680 | 0.7305 | **0.7560** | 24.2220 | 22.8514 | 21.8914 | **20.1930** | 0.6621 | 0.6766 | 0.7593 | **0.7793** |
|  |  | ● | ● | ● | ● | 0.7022 | 0.6727 | 0.7343 | **0.7559** | 22.3157 | 22.2076 | 21.8527 | **18.7885** | 0.6890 | 0.6766 | 0.7573 | **0.7754** |
| **Missing 2** | ● | ● | ● |  |  | 0.7000 | 0.6481 | 0.7396 | **0.7527** | 22.6678 | 22.2320 | 21.4045 | **19.3788** | 0.6940 | 0.6659 | 0.7563 | **0.7735** |
|  | ● | ● |  | ● |  | 0.5158 | 0.5719 | 0.6344 | **0.6634** | 26.1244 | **21.7288** | 24.5996 | 22.9952 | 0.5046 | 0.5699 | 0.6529 | **0.6666** |
|  | ● | ● |  |  | ● | 0.4526 | 0.5557 | 0.6187 | **0.6611** | 34.1281 | **23.5872** | 26.1812 | 25.3373 | 0.4371 | 0.5520 | 0.6412 | **0.6698** |
|  | ● |  | ● | ● |  | 0.6573 | 0.6621 | 0.7388 | **0.7539** | 27.7666 | 21.5190 | 22.3631 | **20.3400** | 0.6385 | 0.6759 | 0.7549 | **0.7739** |
|  | ● |  | ● |  | ● | 0.6942 | 0.6361 | 0.7281 | **0.7522** | 21.6251 | 23.3091 | 20.5818 | **19.7330** | 0.6901 | 0.6613 | 0.7478 | **0.7751** |
|  | ● |  |  | ● | ● | 0.4268 | 0.5895 | 0.6151 | **0.6524** | 25.3513 | **22.9444** | 25.9775 | 24.1961 | 0.4648 | 0.5710 | 0.6486 | **0.6717** |
|  |  | ● | ● | ● |  | 0.6889 | 0.6584 | 0.7321 | **0.7460** | 22.2303 | 22.8942 | 21.4670 | **20.1067** | 0.6744 | 0.6695 | 0.7420 | **0.7638** |
|  |  | ● | ● |  | ● | 0.6936 | 0.6628 | 0.7376 | **0.7537** | 25.1017 | 22.4126 | 22.0546 | **19.3715** | 0.6691 | 0.6728 | 0.7615 | **0.7771** |
|  |  | ● |  | ● | ● | 0.4321 | 0.5491 | 0.6196 | **0.6529** | 26.7032 | **21.5382** | 25.1835 | 23.8236 | 0.4156 | 0.5493 | 0.6488 | **0.6578** |
|  |  |  | ● | ● | ● | 0.5346 | 0.6565 | 0.7377 | **0.7557** | 30.5206 | 22.2408 | 22.2118 | **19.6195** | 0.5337 | 0.6641 | 0.7617 | **0.7770** |
| **Missing 3** | ● | ● |  |  |  | 0.2991 | 0.5428 | 0.5762 | **0.6480** | 37.7770 | **22.2976** | 30.1299 | 27.7272 | 0.3404 | 0.5472 | 0.6455 | **0.6642** |
|  | ● |  | ● |  |  | 0.6780 | 0.6341 | 0.7310 | **0.7496** | **20.5428** | 22.4566 | 22.7195 | 20.8782 | 0.6928 | 0.6597 | 0.7553 | **0.7664** |
|  | ● |  |  | ● |  | 0.3145 | 0.5764 | 0.5510 | **0.6399** | 30.0392 | **21.1060** | 30.9215 | 25.9798 | 0.3798 | 0.5640 | 0.6467 | **0.6648** |
|  | ● |  |  |  | ● | 0.2218 | 0.5114 | 0.4828 | **0.5708** | 31.6513 | **23.2899** | 31.3842 | 30.8125 | 0.2969 | 0.5068 | 0.5708 | **0.6167** |
|  |  | ● | ● |  |  | 0.5975 | 0.6549 | 0.7343 | **0.7418** | 24.7032 | 22.0924 | 21.6443 | **20.7626** | 0.6051 | 0.6705 | 0.7547 | **0.7628** |
|  |  | ● |  | ● |  | 0.1560 | 0.4690 | 0.5205 | **0.6024** | 32.2773 | **21.2130** | 27.2820 | 23.0376 | 0.1851 | 0.5081 | 0.5766 | **0.6207** |
|  |  | ● |  |  | ● | 0.3361 | 0.5289 | 0.5249 | **0.6285** | 36.0531 | **21.9253** | 33.9394 | 29.0944 | 0.3033 | 0.5316 | 0.5992 | **0.6523** |
|  |  |  | ● | ● |  | 0.3500 | 0.6599 | 0.7308 | **0.7421** | 34.0892 | 21.6774 | 21.8857 | **20.3671** | 0.4300 | 0.6700 | 0.7506 | **0.7598** |
|  |  |  | ● |  | ● | 0.5458 | 0.6326 | 0.7290 | **0.7499** | 32.2293 | 22.4765 | 21.2980 | **20.9997** | 0.5208 | 0.6557 | 0.7563 | **0.7684** |
|  |  |  |  | ● | ● | 0.1933 | 0.5512 | 0.5105 | **0.6107** | 30.2241 | **22.0634** | 31.6879 | 26.7575 | 0.2499 | 0.5450 | 0.6183 | **0.6572** |
