# Supplementary material for: Multi-scale Gaussian product-of-experts for missing sequence multiple sclerosis lesion segmentation
Source: Front Radiol. 2026 Sep 29;6:1868811. doi: 10.3389/fradi.2026.1868811 (PMC13658566; doi:10.3389/fradi.2026.1868811)
Supplement: Supplementary file 3 [file Table3.docx]

**Supplementary Table s3. Full quantitative results on MS3SEG**

| **Group** | **Available sequences** | | | **DSC** | | | | **HD95** | | | | **CS** | | | |
| --- | --- | --- | --- | --- | --- | --- | --- | --- | --- | --- | --- | --- | --- | --- | --- |
|  | **T1** | **T2** | **FLAIR** | **HeMIS** | **ModDrop++** | **TC-MoE** | **MG-PoE** | **HeMIS** | **ModDrop++** | **TC-MoE** | **MG-PoE** | **HeMIS** | **ModDrop++** | **TC-MoE** | **MG-PoE** |
| **Full Sequence** | ● | ● | ● | 0.6962 | 0.7360 | 0.7449 | **0.7850** | **21.8544** | 24.1704 | 22.0122 | 23.6660 | 0.7036 | 0.7316 | 0.7518 | **0.8141** |
| **Missing 1** |  | ● | ● | 0.6657 | 0.7217 | 0.7336 | **0.7870** | 23.6363 | 24.2169 | 23.6780 | **23.1771** | 0.6764 | 0.7173 | 0.7408 | **0.8097** |
|  | ● |  | ● | 0.6863 | 0.7332 | 0.7387 | **0.7871** | **22.4678** | 24.0572 | 22.7749 | 23.9105 | 0.6949 | 0.7304 | 0.7467 | **0.8099** |
|  | ● | ● |  | 0.4377 | 0.5378 | 0.5278 | **0.5785** | 24.7510 | **23.7263** | 27.0566 | 25.6950 | 0.4527 | 0.5628 | 0.5403 | **0.6329** |
