# Supplementary material for: Multi-scale Gaussian product-of-experts for missing sequence multiple sclerosis lesion segmentation
Source: Front Radiol. 2026 Sep 29;6:1868811. doi: 10.3389/fradi.2026.1868811 (PMC13658566; doi:10.3389/fradi.2026.1868811)
Supplement: Supplementary file 4 [file Table4.docx]

**Supplementary table s4. Full quantitative results on MSLesSeg24**

| **Group** | **Available sequences** | | | **DSC** | | | | **HD95** | | | | **CS** | | | |
| --- | --- | --- | --- | --- | --- | --- | --- | --- | --- | --- | --- | --- | --- | --- | --- |
|  | **T1** | **T2** | **FLAIR** | **HeMIS** | **ModDrop++** | **TC-MoE** | **MG-PoE** | **HeMIS** | **ModDrop++** | **TC-MoE** | **MG-PoE** | **HeMIS** | **ModDrop++** | **TC-MoE** | **MG-PoE** |
| **Full Sequence** | ● | ● | ● | 0.3971 | 0.4512 | 0.5513 | **0.5566** | 41.6586 | 39.5756 | 32.8627 | **27.3729** | 0.4227 | 0.4884 | **0.6355** | 0.6180 |
| **Missing 1** | ● | ● |  | 0.1095 | 0.1976 | 0.3086 | **0.3449** | 47.5385 | 44.0287 | 39.6411 | **35.7635** | 0.1510 | 0.2537 | 0.3802 | **0.4391** |
|  | ● |  | ● | 0.4650 | 0.4611 | 0.5754 | **0.5951** | 40.6095 | 35.2218 | 33.2618 | **28.1177** | 0.4811 | 0.5185 | 0.6445 | **0.6603** |
|  |  | ● | ● | 0.3251 | 0.4829 | **0.5911** | 0.5888 | 40.9773 | 33.0967 | 30.8800 | **27.6349** | 0.3944 | 0.5292 | **0.6463** | 0.6440 |
